# Criminal Negligence

**Published:** 1875-04

**Authors:** 


The Bistoury.

ELMIRA, N. Y., APRIL, 1875.

The BISTOURY is published Quarterly, upon the 1st of April, July, October and January, at Fifty Cents a year, in advance.

### CRIMINAL NEGLIGENCE.

The reckless negligence that pervades all classes of community relative to the management of the fevers common to children, is so marked as to call for such legislation as will compel physicians and parents to exercise more care in preventing the spread of such highly contagious diseases as scarlet fever, measles, erysipelas, etc.

When a case of smallpox occurs in any community, immediate measures are taken to isolate the patient, and to prevent people from coming to or leaving the house, in order to confine the disease to that one family and preventing the infection of an entire neighborhood. So careful are people in avoiding a case of smallpox, that they will walk squares out of their way, not to pass near the house where the contagion is; yet, when a child is taken with scarlet fever, thoughtless and meddlesome women will "run in, just to talk over the matter" with the mother, and perhaps advise the administration of hot tea, to prevent the rash from "striking in," or some equally ridiculous "remedy," and then rush into a neighbor's dwelling, where there may be a bevy of children, and infect every one of them with this horrible disease.

A child, upon exhibiting the first symptoms of scarlet fever, measles, or diphtheria, should be placed in an upper room, entirely removed from the balance of the family, and one person, naturally the mother, appointed to wait upon and nurse it to healthfulness. Not another individual should be permitted to enter the room, save the family physician, and he should make as short a call as possible, preparing his medicines and giving his advice in the hall or the next room. Before entering another house, he should thoroughly ventilate his clothing by a long ride in the open air, or make his scarlet-fever patient the last one to be visited for the day. Placards should be exhibited upon the doors, announcing the character of the disease, as is done in smallpox, and visitors absolutely prohibited from entering the house. If such quarentine regulations should be enforced in every case, during the period that desquamation is occurring—that is, until all the little scales have *entirely and completely fallen from the person*, we would never hear of the prevalence of scarlet fever and measles, and the terrible sequalæ that usually accompany these diseases; for, be it known, that, of the two diseases, scarlet fever and smallpox, we consider, as do all physicians, the latter the least dangerous, and the least serious in its consequences.

Another abominable habit among women, is to kiss, upon the lips, every little child they chance to meet. Should the lady have a sore throat, she is apt to communicate it to the child; or should the child have a diphtheritic throat, the poison will rest upon the lips of the lady to be communicated to the next innocent one upon whom she afflicts her caress. Entire neighborhoods of children have been poisoned in this manner, by one thoughtless woman.

When it is known that scarlet fever, measles, erysipelas, and diphtheria, are as contagious, and *much more dangerous* and serious in their consequences, than smallpox, perhaps, the people will be awakened to the fact that it is quite as important to isolate the cases as completely as is done in the last mentioned contagion. Otherwise, our legislators should *compel* such carefulness upon the part of physicians and parents as will prevent the distributing of these terrible diseases.

